# Radiologist versus Non-Radiologist Detection of Lymph Node Metastasis in Papillary Thyroid Carcinoma by Ultrasound: A Meta-Analysis

**DOI:** 10.3390/biomedicines10102575

**Published:** 2022-10-14

**Authors:** Peter P. Issa, Lauren Mueller, Mohammad Hussein, Aaron Albuck, Mohamed Shama, Eman Toraih, Emad Kandil

**Affiliations:** 1 School of Medicine, Louisiana State University Health Sciences Center, New Orleans, LA 70112, USA; 2 School of Medicine, Tulane University, New Orleans, LA 70112, USA; 3 Department of Surgery, School of Medicine, Tulane University, New Orleans, LA 70112, USA; 4 Genetics Unit, Department of Histology and Cell Biology, Faculty of Medicine, Suez Canal University, Ismailia 41522, Egypt

**Keywords:** thyroid cancer, ultrasound, lymph node metastasis, radiologist

## Abstract

Papillary thyroid carcinoma (PTC) is the most common thyroid cancer worldwide and is known to spread to adjacent neck lymphatics. Lymph node metastasis (LNM) is a known predictor of disease recurrence and is an indicator for aggressive resection. Our study aims to determine if ultrasound sonographers’ degree of training influences overall LNM detection. PubMed, Embase, and Scopus articles were searched and screened for relevant articles. Two investigators independently screened and extracted the data. Diagnostic test parameters were determined for all studies, studies reported by radiologists, and studies reported by non-radiologists. The total sample size amounted to 5768 patients and 10,030 lymph nodes. Radiologists performed ultrasounds in 18 studies, while non-radiologists performed ultrasounds in seven studies, corresponding to 4442 and 1326 patients, respectively. The overall sensitivity of LNM detection by US was 59% (95%CI = 58–60%), and the overall specificity was 85% (95%CI = 84–86%). The sensitivity and specificity of US performed by radiologists were 58% and 86%, respectively. The sensitivity and specificity of US performed by non-radiologists were 62% and 78%, respectively. Summary receiver operating curve (sROC) found radiologists and non-radiologists to detect LNM on US with similar accuracy (*p* = 0.517). Our work suggests that both radiologists and non-radiologists alike detect overall LNM with high accuracy on US.

## 1. Introduction

Thyroid cancer is the most common endocrine malignancy [1]. Papillary thyroid carcinoma (PTC) is the most common thyroid cancer, accounting for 90% of thyroid cancer diagnoses [2,3]. Though sometimes described as an indolent disease, PTC often metastasizes into adjacent neck lymphatics, increasing the risk of disease recurrence [4]. The risk of PTC recurrence can be as high as 30% [5], and accordingly the determination of factors that can predict PTC aggressiveness is critical. One known independent risk factor for local PTC recurrence is lymph node metastasis (LNM) [5,6].

Cervical LNM is found in 20–50% of PTC patients [7]. Most commonly, lymphatic spread carries PTC metastases toward the central compartment of the neck (level VI). Metastasis is directly related to recurrence and mortality, making an accurate and efficient diagnosis of LMN paramount in managing these patients [8,9]. Furthermore, both the American Thyroid Association (ATA) and the British Thyroid Association (BTA) guidelines often recommend prophylactic central neck dissection with total thyroidectomy as the standard for operative PTC management [10,11]. Current ATA guidelines recommend ultrasound (US) as the first-line diagnostic technique in assessing LMN in PTC patients [11]. Though US is widely available and considerably cheaper than other imaging modalities, US diagnosis is operator-dependent and has been demonstrated to possess variable sensitivity and specificity [12,13]. Additionally, retrosternal, retropharyngeal, and mediastinal visualization may be challenging depending on the sonographer’s level of training [14]. Importantly, accurate lymph node mapping allows for a targeted surgical approach, minimizing the area of neck dissection, decreasing mortality, and increasing optimal cosmetic outcomes [15].

Since US is common practice for patients with thyroid disease and yet an operator-dependent technique, we thought to investigate the diagnostic value of US imaging performed by a radiologist, as opposed to performed by a non-radiologist, such as an ultrasound technician or surgeon.

## 2. Methods

This systematic review and meta-analysis followed the Preferred Reporting Items for Systematic Review and Meta-Analyses (PRISMA) guidelines for diagnostic test accuracy [16].

### 2.1. Literature Search

Multiple databases were searched in this meta-analysis including PubMed, Embase, and Scopus. The search terms were as follows: “thyroid” AND “lymph node metastasis” OR “LNM” AND “ultrasonography” OR “sonography” OR “ultrasound” OR “US.” The search was performed on April 2022 and conducted without time or language restriction. All abstracts and the subsequent full texts were screened to determine the final articles.

### 2.2. Inclusion and Exclusion Criteria

Studies included in our analysis were those which were (1) cohort studies, case controls, or randomized controlled trials, (2) reporting pertinent parameters with respect to LNM detection on preoperative US in PTC patients, (3) confirmed by surgical pathology for the presence and/or absence of LNM. Importantly, each study must have reported sonographer qualification and diagnostic performance metrics such as sensitivity and specificity (or at least calculatable). Works reporting unoriginal work or not of the above-mentioned study types were excluded, including letters, opinions, editorials, case reports, singular abstracts, and reviews.

### 2.3. Data Extraction

Eligible articles were screened and reviewed by two investigators (P.P.I. and A.A.) and subsequently extracted. Any inconsistencies were settled by a senior author. Data relevant to the study were extracted, including study characteristics such as author, publication year, country and institution, study design, study period, and sample size. Importantly, the sonographer and their level of radiologic training (radiologist, US technician, or surgeon) and the number of lymph nodes detected were also included. Sensitivity and specificity were either extracted or manually calculated from the number of true positives, false positives, true negatives, and false negatives.

### 2.4. Statistical Analysis

Statistical analysis was performed using MetaDisc1.4 software (Unit of Clinical Biostatistics, Madrid, Spain) [17]. Sensitivity, specificity, likelihood ratios (LR), diagnostic odds ratio (DOR), and a 95% confidence interval (CI) were calculated. The area under the curve (AUC) was estimated for each group. Diagnostic accuracy measures were compared between studies with data recorded by radiologists and those by non-radiologists (US technicians and surgeons) using a student’s *t*-test.

We quantified the heterogeneity using the I-square (I^2^) and Chi-squared tests. A fixed-effects model was used to analyze the selected studies’ consistency (I^2^ < 50% and *p* > 0.05). If I^2^ > 50% or *p* < 0.05, heterogeneity was present. Pooled estimates were performed using the random effects model if there was no obvious reason for heterogeneity. Possible heterogeneity caused by the threshold effect was tested. If there is a strong positive correlation between the logit of sensitivity and logit of 1-specificity (*p* < 0.05), assessed by Spearman’s correlation coefficients, threshold effects were present. Furthermore, meta-regression models were conducted to trace other heterogeneity sources according to the study design (retrospective versus prospective), sample size (400 patients or more versus less than 400), and year of publication (published in 2015 or more recently versus before). Regression diagnostic odds ratios (rDOR) were reported.

## 3. Results

### 3.1. Literature Search

**Figure 1:**
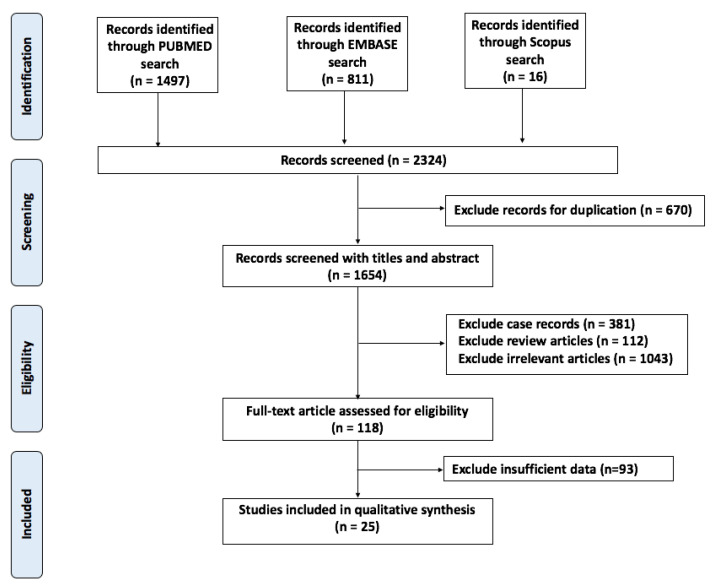
Workflow of the literature search. A total of 25 studies were included.

A total of 1654 unique articles (2423 total, 670 duplicates) were found using search terms previously mentioned. We excluded 1536 articles as they did not meet inclusion criteria. The remaining articles were reviewed in-depth and considered until the final number of studies was reached. A total of 25 studies were included in our meta-analysis. All studies were published between 2007 and 2022 and include works from Korea (11 studies), China (7 studies), the United States (5 studies), and Chile (1 study) [13,18,19,20,21,22,23,24,25,26,27,28,29,30,31,32,33,34,35,36,37,38,39,40]. Four works were published within the last two years (2020 and beyond), suggesting heightened interest in determining the accuracy of diagnostic imaging. The workflow of the literature search is depicted in Figure 1.

### 3.2. Characteristics of the Study Population

The characteristics of the included studies are detailed in Table 1. Of 25 studies, 17 were retrospective in study design and eight were prospective. The overall study period included patients from 1993 to 2022. The total sample size consisted of 5768 patients with 10,030 lymph nodes analyzed. Radiologists performed ultrasounds in 18 studies, while non-radiologists performed ultrasounds in 7 studies, corresponding to 4442 and 1326 patients, respectively. All LNM diagnoses were confirmed by surgical pathology.

### 3.3. Detection Accuracy of Lymph Node Metastasis Overall

The overall sensitivity of LNM detection by US was 59% (95%CI = 58–60%) and the overall specificity was 85% (95%CI = 84–86%) (Table 2). The corresponding positive and negative LRs were 3.66 (95%CI = 2.88–4.66) and 0.49 (95%CI = 0.42–0.57), respectively. The DOR was 8.04 (95%CI = 5.86–11.03) and the AUC was determined to be 0.800 ± 0.022.

### 3.4. Detection of Lymph Node Metastasis Sub-Grouped by Operator

**Figure 2:**
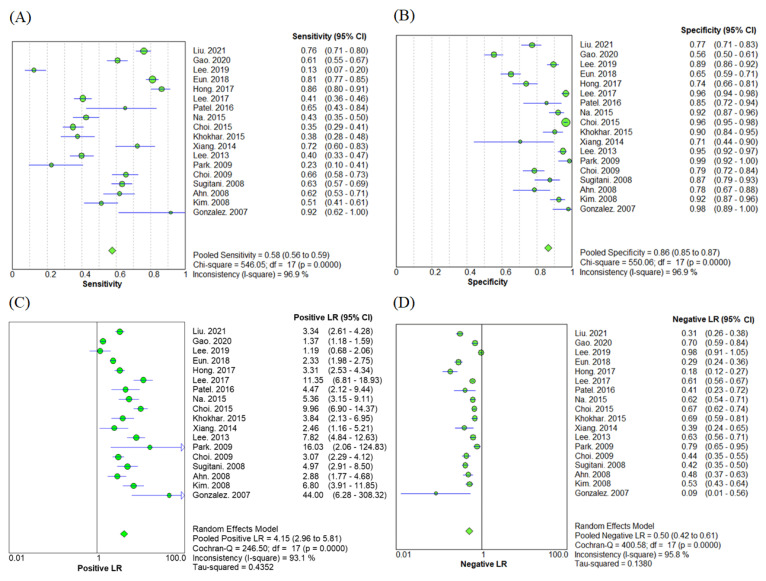
Diagnostic parameters of ultrasound performed by radiologists [18,20,22,23,24,25,26,27,28,29,30,32,34,35,36,37,38,39]. The (**A**) sensitivity, (**B**) specificity, (**C**) positive likelihood ratio, and (**D**) negative likelihood ratio are presented. Data is reported as an estimate and 95% confidence interval. Heterogeneity was assessed using Cochran-Q test and magnitude was estimated by the I-square value. CI = Confidence Interval; LR = Likelihood Ratio.

A total of 18 studies reported the diagnostic accuracy of LNM detection by US performed by radiologists. This study population included 4442 patients and 7920 lymph nodes. The sensitivity and specificity of US detection of LNM by radiologists were 58% (95%CI = 56–59%) and 86% (95%CI = 85–87%), respectively (Figure 2A,B). The positive LR was 4.15 (95%CI = 2.96–5.81) and the negative LR was 0.50 (95%CI = 0.42–0.61) (Figure 2C,D).

**Figure 3:**
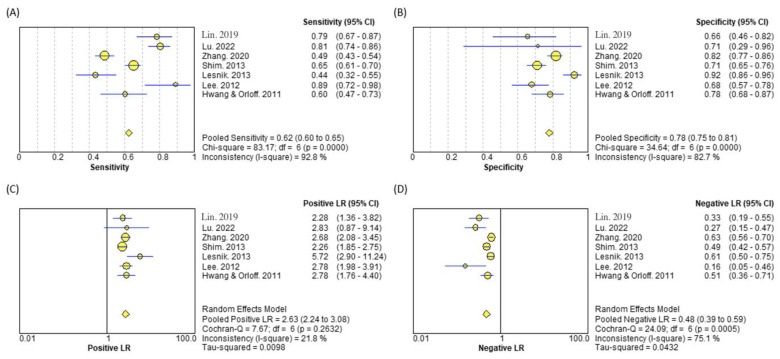
Diagnostic parameters of ultrasound performed by non-radiologists [13,19,21,22,31,33,41]. The (**A**) sensitivity, (**B**) specificity, (**C**) positive likelihood ratio, and (**D**) negative likelihood ratio are presented. Data is reported as an estimate and 95% confidence interval. Heterogeneity was assessed using Cochran-Q test and magnitude was estimated by the I-square value. CI = Confidence Interval; LR = Likelihood Ratio.

US detection of LNM by non-radiologists (surgeon or US technician) was reported in 7 studies, totaling 1326 patients and 2110 lymph nodes. Pooled estimates of sensitivities and specificities were 62% (95%CI = 60–65%) and 78% (95%CI = 75–81%), respectively (Figure 3A,B). The positive LR was 2.63 (95%CI = 2.24–3.08), and the negative LR was 0.48 (95%CI = 0.39–0.59) (Figure 3C,D).

**Figure 4:**
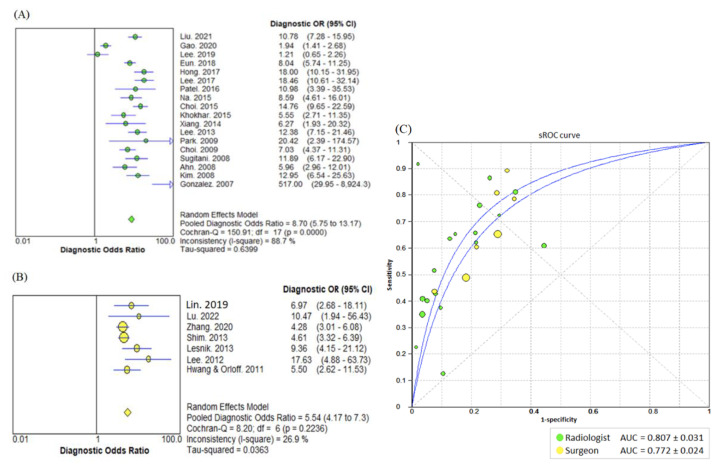
Comparison of diagnostic parameters of ultrasound performed by radiologists with those performed by non-radiologists. The (**A**) diagnostic odds ratio of radiologists [18,20,23,24,25,26,27,28,29,30,32,34,35,36,37,38,39], (**B**) diagnostic odds ratio of non-radiologists [13,19,21,22,31,33,41], and (**C**) summary receiver operating curve are presented. Summary receiver operating curve is presented as an area under the curve and standard error. Heterogeneity was assessed using Cochran-Q test and magnitude was estimated by the I-square value. OR = Odds Ratio; CI = Confidence Interval; AUC = area under the curve; sROC = summary receiver operating curve.

The DOR of LNM detection was 8.70 (95%CI = 5.75–13.17) for radiologists and 5.54 (95%CI = 4.17–7.35) for non-radiologists. Comparing the two groups, the AUC was found to be similar at 0.806 ± 0.031 for radiologists and 0.773 ± 0.024 for non-radiologists (*p* = 0.517) (Figure 4).

### 3.5. Heterogeneity Analysis

Apart from positive LR (I^2^ = 21.8%, *p* = 0.26) and DOR (I^2^ = 26.9%, *p* = 0.22) in readings by radiologists, all analyses displayed significant heterogeneity (Supplementary Table S1). To trace this heterogeneity, analysis of the diagnostic threshold showed significant correlation (spearman correlation) between the true and false positive rates in LNM detection in our overall analysis (r = 0.619, *p* = 0.001), subgroup analysis for radiologists (r = 0.528, *p* = 0.021), and subgroup analysis for non-radiologists (r = 0.821, *p* = 0.023) (Supplementary Figure S1).

After adjustment of study covariates including study design, US operator, sample size, and the year of publication, meta-regression analysis did not show any significant results. The rDOR based on the sonographer was 1.15 (95%CI = 0.77–1.72; *p* = 0.48) (Supplementary Table S2).

## 4. Discussion

Preoperative assessment of LNM in PTC patients is imperative in surgical planning and therefore directly impacts patient outcomes. Importantly, malignancy staging (i.e., TNM staging) is often more important than malignancy grading in determining patient prognosis. Since US is the most common and widely available imaging technique for the thyroid gland, its diagnostic accuracy, including sensitivity, specificity, DOR, and AUC has been studied extensively [33,42]. Yet, to our best knowledge, this is the first meta-analysis to investigate the diagnostic accuracy of overall LNM detection by US performed by radiologists and non-radiologists.

Ultrasound is currently the gold standard and first line of practice in preoperative assessment of PTC and in detecting LNM. It is easy to perform, widely available, low cost, and safe with no risk of radiation [43]. Previous studies have consistently determined sensitivity and specificity reports between 30–57% and 82–92%, respectively [33,44,45]. Our work corroborates the current literature, finding the overall sensitivity and specificity of LNM detection by US to be 59% and 85%, respectively. While subsequent sub-group analyses for central and lateral compartment LNM in our study is warranted, we found that further sub-stratification limited the patient cohorts significantly. Since LNM is an important independent predictor of patient prognosis, several studies have suggested adjunct imaging modalities, such as computed tomography (CT), to help improve the low sensitivity often seen in US to minimize the missed detection of true positives [46,47]. Though prophylactic lymph node dissection during thyroidectomy has been debated [48,49,50,51], complete and thorough surgical resection can positively affect patient survival [52,53]. In consequence, determining factors which could optimize the accuracy of LNM detection, such as the impact of US operator, is important in improving patient outcomes.

Traditionally, radiologists performed ultrasound prior to operative management. However, there has been a movement towards surgeon-preformed ultrasound as an extension of their operative management [54,55]. Oltmann et al. found that surgeons documented lymph node status more often than radiologists and that surgeon-performed US patients had less disease recurrence (0% versus 12%, *p* = 0.01) [56]. With respect to ultrasound-guided thyroid fine-needle aspiration (FNA), Graciano et al. reported no difference in efficacy when performed by radiologists or non-radiologists [57]. Other studies demonstrate that experience greater than seven years increases the positive predictive value and confidence of LNM detection [58]. While other meta-analyses have shown differences in diagnostic testing accuracy of US versus CT staging, our study is the first to search and analyze the literature for differences in US staging performed by radiologists compared to non-radiologists [45,59]. Our work found that US performed by radiologists were of similar diagnostic accuracy (*p* = 0.517) as those performed by non-radiologists. Though our study analyzes over 10,000 lymphatic nodules, our findings are not generalizable to the analysis of other cancers and warrants further study, such as randomized controlled trials, to further determine the role of US technicians in the non-radiologist grouping.

Detection of LNM on preoperative evaluation is critical in patients undergoing active surveillance management. Active surveillance management is the careful monitoring of patients with low-risk primary PTC (small size or without suspicious US features) by yearly or bi-yearly US and/or CT to detect thyroid nodule changes [60]. Importantly, LNM detection is an indication to immediately terminate active surveillance management and proceed with surgical treatment. Since early detection of cancer is vital in patient prognosis and allowing patients to maintain active surveillance management can increase mortality by as much as 130% [5], it is imperative that US diagnostic accuracy be optimized. Further work should elucidate other potentially-relevant factors such as patient body habitus [28].

Finally, we acknowledge several limitations of this study. Although the large sample size allowed for robust analyses, the majority of studies included were retrospective. Furthermore, sub-group analysis by cervical compartment was not feasible, as the four-way split (radiologist vs. non-radiologist as well as central vs. lateral LNM) limited the study population significantly. Additionally, studies took place in multiple countries. While this may lead to a more diverse patient population and greater generalizability, different training qualifications may exist for radiologists, US technicians, and surgeons across locations, which may provide for difficulty in comparing diagnostic testing parameters. In addition, the limited number of studies reporting US performance metrics of surgeons necessitated a non-radiologist versus radiologist comparison. Future works should look to determine the influence of surgeons and US technicians alone, as their trainings differ significantly. Another limitation in our study is the lack of studies reporting the readings of endocrinologists, leaving their detection accuracy unexplored. Finally, whether our findings are consistent with other cancer imaging studies is unknown and warrants further investigation.

## 5. Conclusions

Diagnostic accuracy of LNM detection on US performed by radiologists and non-radiologists were similar. Our work suggests that both radiologists and non-radiologists alike detect LNM with high accuracy on US.

## Figures and Tables

**Table 1:** Characteristics of the study population.

First Author	Year	Country	Study Period	Study Design	Sample Size	Number of Lymph Nodes	Sonographer Qualification
Lu [19]	2022	China	01/2018–01/2020	Retro	185	185	US Technician
Liu [18]	2021	China	04/2016–05/2018	Retro	600	661	Radiology
Gao [20]	2020	China	05/2017–07/2017	Pro	501	618	Radiology
Zhang [21]	2020	China	01/2018–09/2018	Retro	665	665	US Technician
Li [40]	2019	China	01/2017–12/2017	Pro	30	99	US Technician
Lee [22]	2019	Korea	12/2014–12/2016	Retro	218	479	Radiology
Eun [23]	2018	Korea	01/2013–12/2015	Retro	302	1128	Radiology
Hong [24]	2017	China	01/2014–09/2016	Pro	253	319	Radiology
Lee [25]	2017	Korea	11/2011–12/2012	Pro	351	801	Radiology
Patel [26]	2016	USA	01/2011–12/2011	Retro	44	71	Radiology
Na [27]	2015	Korea	03/2011–02/2012	Retro	176	352	Radiology
Choi [28]	2015	Korea	12/2012–04/2013	Retro	625	1250	Radiology
Khokhar [29]	2015	USA	2004–2014	Retro	51	227	Radiology
Xiang [30]	2014	China	05/2012–01/2014	Retro	53	82	Radiology
Shim [31]	2013	Korea	2006–2010	Retro	143	715	US Technician
Lee [32]	2013	Korea	01/2007–5/2010	Retro	252	558	Radiology
Lesnik [13]	2013	USA	2003–2008	Pro	95	196	US Technician
Lee [41]	2012	USA	2000–2008	Retro	109	109	Endocrine Surgeon
Hwang & Orloff [33]	2011	USA	10/2004–12/2007	Retro	99	141	Head and Neck Surgeon
Park [34]	2009	Korea	11/2006–03/2007	Pro	94	147	Radiology
Choi [39]	2009	Korea	02/2006–04/2007	Retro	299	352	Radiology
Sugitani [35]	2008	Japan	1993–2001	Pro	361	357	Radiology
Ahn [36]	2008	Korea	01/2005–12/2005	Retro	37	181	Radiology
Kim [37]	2008	Korea	04/2006–10/2006	Retro	165	277	Radiology
Gonzalez [38]	2007	Chile	01/2006–06/2006	Pro	60	60	Radiology

In the study design column, “Pro” designates prospective, and “Retro” designates retrospective. US = Ultrasound.

**Table 2:** Diagnostic accuracy of overall lymph node metastasis detection by ultrasound by radiologists or non-radiologists.

Diagnostic Parameter	Meta-Analysis Pooled Estimate (95%CI)
Sensitivity	59% (58–60%)
Specificity	85% (84–86%)
Positive Likelihood Ratio	3.66 (2.88–4.66)
Negative Likelihood Ratio	0.49 (0.42–0.57)
Diagnostic Odds Ratio	8.04 (5.86–11.03)
Area Under the Curve (SE)	0.800 ± 0.022

A total of 25 studies were included in our meta-analysis. Total sample size consisted of 5768 patients with 10,030 lymph nodes analyzed. Radiologists performed ultrasounds in 18 studies, while non-radiologists performed ultrasounds in 7 studies, corresponding to 4442 and 1326 patients, respectively. CI = Confidence Interval; SE = Standard Error.

## Data Availability

Data is contained within the article or Supplementary Material.

## Supplementary Materials

The following supporting information can be downloaded at: https://www.mdpi.com/article/10.3390/biomedicines10102575/s1, Table S1: Heterogeneity analysis; Table S2: Metaregression analysis of parameters possibly influencing ultrasound diagnostic testing accuracy; Figure S1: Analysis of diagnostic threshold. Spearman correlation analysis for Logit (TPR) vs. Logit (FPR) was employed. Black: correlation coefficient of the overall analysis, Green: radiologist operator studies, and Orange: non-radiologist operator studies.
