# Supplementary material for: Radiologist versus Non-Radiologist Detection of Lymph Node Metastasis in Papillary Thyroid Carcinoma by Ultrasound: A Meta-Analysis
Source: Biomedicines. 2022 Oct 14;10(10):2575. doi: 10.3390/biomedicines10102575 (PMC9599420; doi:10.3390/biomedicines10102575)
Supplement: Supplementary file 1 [file biomedicines-10-02575-s001.zip › biomedicines-1917324-supplementary.pdf]

Supplementary Table S1. Heterogeneity analysis.

	Overall		Radiologist		Non-Radiologist	
**Diagnostic Parameter**	**I^2**	***p*-value**	**I^2**	***p*-value**	**I^2**	***p*-value**
Sensitivity	96.2%	<0.001	96.9%	<0.001	92.8%	<0.001
Specificity	96.1%	<0.001	96.9%	<0.001	82.7%	<0.001
Positive Likelihood Ratio	90.4%	<0.001	93.1%	<0.001	21.8%	0.26
Negative Likelihood Ratio	94.4%	<0.001	95.8%	<0.001	75.1%	<0.001
Diagnostic Odds Ratio	85.7%	<0.001	88.7%	<0.001	26.9%	0.22

Supplemental Table S2. Metaregression analysis of parameters possibly influencing ultrasound diagnostic testing accuracy.

Parameter	*p*-value	rDOR	95%CI
Sonographer (radiologist vs. non-radiologist)	0.48	1.15	(0.77 – 1.72)
Study design (retrospective vs. prospective)	0.19	0.60	(0.28 – 1.31)
Sample size (>400 vs. ≤400 patients)	0.82	1.09	(0.49 – 2.46)
Publication year (>2015 vs ≤2015)	0.16	0.57	(0.25 – 1.28)

rDOR = regression diagnostic odds ratio; CI = Confidence Interval.

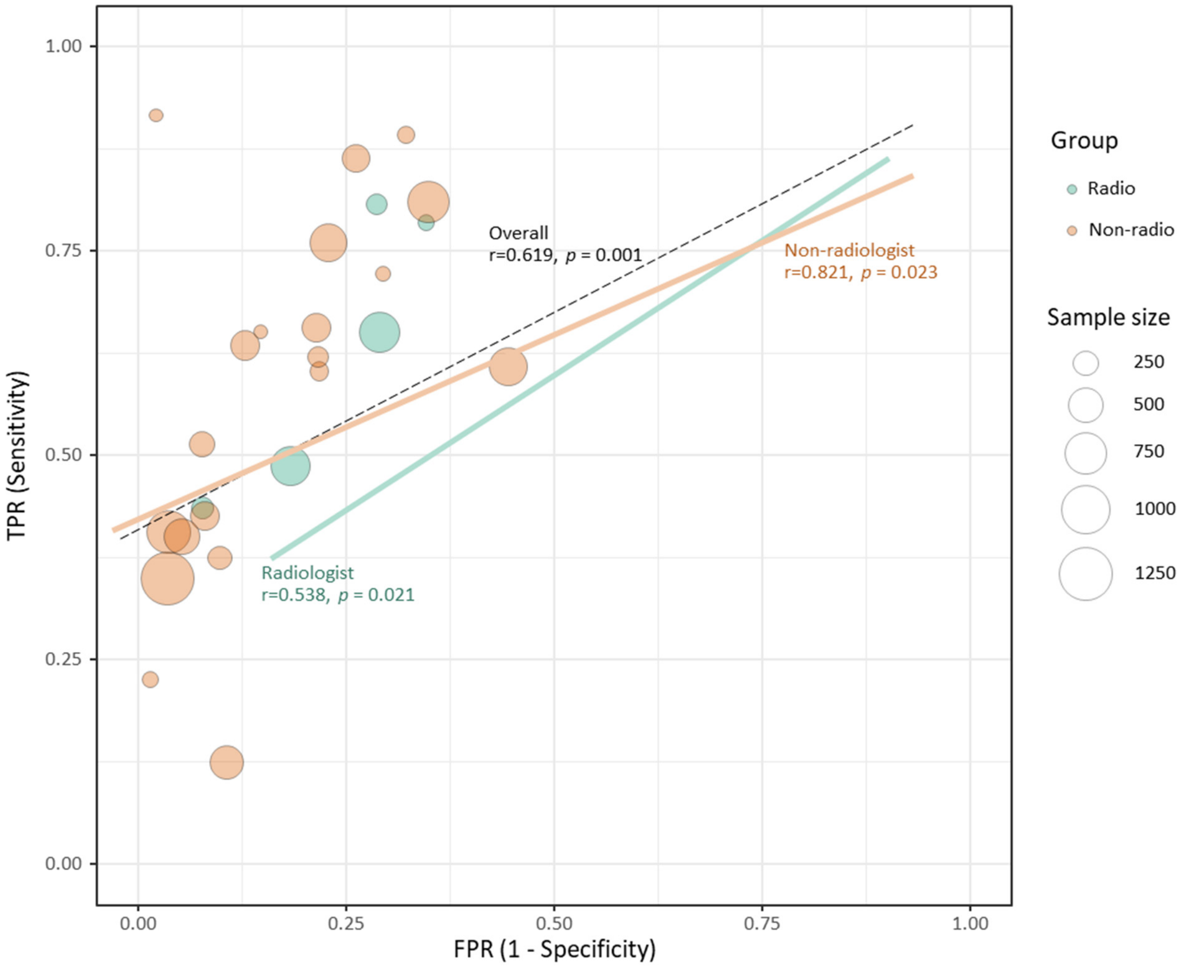

Supplemental Figure S1. Analysis of diagnostic threshold. Spearman correlation analysis for Logit (TPR) vs. Logit (FPR) was employed. Black: correlation coefficient of the overall analysis, Green: radiologist operator studies, and Orange: non-radiologist operator studies.
